# HIV Testing and Diagnosis Rates in Kiev, Ukraine: April 2013 - March 2014

**DOI:** 10.1371/journal.pone.0137062

**Published:** 2015-08-31

**Authors:** Ruth Simmons, Ruslan Malyuta, Nelli Chentsova, Antonia Medoeva, Yuri Kruglov, Alexander Yurchenko, Andrew Copas, Kholoud Porter, CASCADE Collaboration in EuroCoord

**Affiliations:** 1 MRC Clinical Trials Unit at University College London, London, United Kingdom; 2 Perinatal Prevention of AIDS Initiative, Odessa, Ukraine; 3 Kyiv City AIDS Center, Kyiv, Ukraine; 4 Institute of Epidemiology, Kyiv, Ukraine; Istituto Superiore Sanita', ITALY

## Abstract

**Objective:**

Data from Ukraine on risk factors for HIV acquisition are limited. We describe the characteristics of individuals testing for HIV in the main testing centres of the Ukrainian capital Kiev, including HIV risk factors, testing rates, and positivity rates.

**Methods:**

As part of a larger study to estimate HIV incidence within Kiev City, we included questions on possible risk factors for HIV acquisition and testing history to existing systems in 4 infectious disease clinics. Data were provided by the person requesting an HIV test using a handheld electronic tablet. All persons (≥16yrs) presenting for an HIV test April 2013–March 2014 were included. Rates per 100,000 were calculated using region-specific denominators for Kiev.

**Results:**

During the study period 6370 individuals tested for HIV, equivalent to a testing rate of 293.2 per 100,000. Of these, 467 (7.8%) were HIV-positive, with the highest proportion positive among 31–35 year olds (11.2%), males (9.4%), people who inject drugs (PWID) (17.9%) and men who have sex with men (MSM) (24.1%). Using published population size estimates of MSM, diagnosis rates for MSM ranged from 490.6to 1548.3/100,000. A higher proportion of heterosexual women compared to heterosexual men reported contact with PWID, (16% vs. 4.7%) suggesting a bridging in risk between PWID and their sexual partners.

**Conclusion:**

Collection of HIV risk factor information in Kiev, essential for the purposes of developing effective HIV prevention and response tools, is feasible. The high percentage of MSM among those testing positive for HIV, may indicate a significant level of undisclosed sex between men in national figures.

## Introduction

At 37.1 per 100,000, Ukraine has one of the highest HIV diagnosis rates in Europe during 2012 [1] and estimated adult prevalence of 1.1% [2]. Although the first HIV report was in 1987, the majority of diagnoses of HIV were made from 1995 onwards [3] and, by 2009, the number diagnosed was more than double that of 2001 [2], with yearly diagnosis rates rising from 12.5 per 100,000 population in 2001 [4] to 37.1 in 2012 [1].

Instrumental to this rapid increase was injecting drug use, with outbreaks of HIV initially identified in the southern regions of Odessa and Mykolaiv among people who inject drugs (PWID) [5–7]. However, in more recent years HIV infection attributable to sexual transmission has increased with the number of diagnoses reported to be due to sex between men and women overtaking that among PWID [3]. A suggested reason for this increase is the bridging between PWID and the heterosexual population through male injecting drug users transmitting to their female sexual partners [8].

Furthermore, men who have sex with men (MSM) and commercial sex workers (CSW) [9] are part of a *‘hidden epidemic’*. In particular, MSM as a group, are subject to stigma and discrimination [3, 10], with Ukraine recently considering following the Russian anti-propaganda bill against distributing information on non-traditional sexual attitudes [11, 12]. Such acts make MSM less likely to identify as such [9], making appropriate targeted public health interventions problematic. Based on published literature, including the European MSM internet survey (EMIS) (0.3%) [13], and work by AIDS Alliance using a social network scale-up method, population surveys and estimates from other countries (0.9%) [14], estimates of MSM living in Ukraine ranged from 62,888 to 198,470. Further, a sentinel study in 2011 interviewing 5950 MSM from all regions of Ukraine, estimated an overall prevalence of 6.4%, ranging from 2% in Mykolayiv to 20% in Donetsk [15]. Yet only 152 of 16,872 new HIV diagnoses in 2012 were reported to be among MSM [1], a considerable under-estimate if the aforementioned figures are to be believed.

In line with WHO testing guidelines [16], free and confidential testing for HIV is accessible nationwide, with service-initiated screening also available in antenatal settings, prisons, blood donor facilities and for policy and regulation reasons. In 2012, just over 2 million tests were conducted in Ukraine overall, a 49% increase on the 1.5 million reported in 2004 [1].

In this study, we sought to collect more information than is currently available about persons presenting for an HIV test in the main testing centres of the Ukrainian capital Kiev, to establish the feasibility of collecting such data, and estimate HIV testing rates, positivity rates, and characterise those at risk of HIV. These data are helpful to healthcare planners and, in particular, for the design of appropriately-targeted healthcare interventions. They are also needed as part of a larger study to estimate HIV incidence within that region.

## Methods

In April 2013 we introduced new methodology for data collection of persons presenting for an HIV test at the main testing facilities within the City of Kiev, to characterise populations at risk. These testing facilities consisted of four infectious disease clinics for HIV, collectively known as the Kiev City AIDS Centre.

All adults (≥16years) presenting or referred for an HIV test at the Kiev City AIDS Centre, between April 1^st^ 2013 and March 31^st^ 2014 were included. Persons testing as part of antenatal or blood donation screening were excluded, as different methodologies for estimating HIV incidence are required for these populations, given that population sizes for these groups are known.

At the time of an individual’s initial HIV test, limited information is usually collected by the healthcare provider seeking input from the attendee. Such information included: identification number, year of birth, residence and reason for test, but no information on probable route of exposure is explicitly sought. Working with the non-governmental organisation Perinatal Prevention of AIDS Initiative (PPAI) and the AIDS centre, we introduced a short anonymous questionnaire to be completed at the time of testing by the clinic attendee using a handheld electronic tablet. Medical staff completed year of birth, sex and identification number before passing the electronic tablet to the attendee. A short explanation about the questionnaire was provided and patients were asked to give their consent to continue or to terminate the session. A set of four short questions followed asking information on residence, risk factors for acquiring HIV, reason for test and testing history. The electronic tablet was chosen to ensure persons felt comfortable with disclosing high risk behaviours and maintain anonymity.

### Ethics statement

The study was part of CASCADE within EuroCoord (www.EuroCoord.net) funded by the European Union Framework Programme VII. Ethics approval was given by the ethics committee of the Institute of Epidemiology and Infectious Diseases, Academy of Medical Sciences of Ukraine, Kiev, Ukraine and the ethics committee of University College London (UCL). The study was an extension of the current routine surveillance methods already defined in Kiev City, consent for an HIV test is required from all individuals aged 14 years and over, those under the age of 14 require parental consent. LAg Avidity testing was performed on residual samples and no name, address or otherwise identifiable information was available in the analysis file. Only adults (≥16years) were included within the study, age categories were defined in the project protocol, which was reviewed and received ethics approval, no persons under the age of 16 years were included within the study. Patients did not experience any additional procedures and no additional consent was sought. The short anonymous questionnaire was introduced as routine surveillance to be completed at the time of testing by the clinic attendee using a handheld electronic tablet. A short explanation about the questionnaire was provided and patients consented by completing the questionnaire. If patients did not consent they could terminate the session.

### Data Management

Data were captured by a remote secure server and downloaded for analysis. Records were de-duplicated using year of birth, sex, clinic and identification number, with discrepancies sent back to the laboratory. The identification number was used to match the person’s data with their HIV test result. All records with incomplete information (year of birth, sex and date of diagnosis) or where a previous HIV test was positive, and, therefore, where the diagnosis was not a new one, were removed. Data are presented for persons aged 16 years and over by age quartiles.

Probable route of exposure was determined based on the attendee’s answer to the following questions: ever injected drugs, ever had sex with a person of the opposite sex, ever had sex with a person of the same sex, ever paid for sex, and ever been paid for sex. Persons with more than one reported risk factor were classified by the following hierarchical order: PWID, MSM, then heterosexual contact. Additional information was available on sexual behaviour (ever paid for sex, ever been paid for sex or partner of a PWID).

Reason for test was grouped into the following three categories: clinical indication (persons presenting with symptoms), high risk population (those regarded to engage in high risk behaviour i.e. injecting drugs, contact of a known HIV-positive person, those diagnosed with a sexually transmitted infection, those who experienced an occupational HIV risk, and those reporting sex with multiple partners), and general screening (before surgical interventions, recruitment in to the army, prisoners and persons requiring an HIV test due to regulations or policy, e.g. for employment or visa procurement). Persons with more than one reported reason for test were classified by the following hierarchical order: general screening, clinical indication, and high risk population.

Persons were classified as repeat testers if there was evidence of at least one previous negative HIV test. Persons were excluded if the previous test was within a month of the current test, if the previous test result was not reported or was equivocal, or if no previous test date was reported.

Rates per 100,000 population were calculated for HIV tests and HIV diagnoses. Region specific denominators for Kiev City were based on data collected through the government census [17].

Population data for MSM, were based on work by the AIDS Alliance [14] and the European MSM internet survey (EMIS) [13]. The AIDS Alliance used the method of ‘anonymous friend’, whereby persons were asked to list the behaviours of friends, as well as data from behaviour studies conducted among at risk populations and sociological surveys. EMIS estimated population rates using the diagnosis rate for the Ukraine, the number of survey members, and those survey members diagnosed with HIV.

### Statistical Analysis

Descriptive statistics are given for persons presenting for an HIV test. Using logistic regression models, we investigated the effect of the following variables on being a repeat tester: age, sex, residence (Kiev City, Outside Kiev City), probable route of exposure (heterosexual, MSM, PWID) and reason for test (clinical indicators, general public screening, high risk behaviour). Two-way interactions were examined.

Statistical analyses were performed using STATA version 13 (STATA Corp, College Station, TX, USA). Only samples from individuals aged 16 years and over were included in analysis.

## Results

During the 12-month period April 2013 –March 2014, 6402 persons were tested for HIV. Thirty-two were excluded as follows: 7 records were missing year of birth, sex or identification number, and 25 persons were already known to be HIV positive.

The remaining 6370 tests (3425 in men and 2945 in women) gave a crude test rate of 293.2 per 100,000 population (aged ≥16 years) (Table 1). The median age at test was 30 years (IQR: 26–36), with the majority seeking testing due to symptoms. HIV test results could not be linked for 353 persons. Of the remaining 6017 tests with a result, 467 (7.8%) were HIV positive, equivalent to a diagnosis rate of 21.5 per 100,000 persons (Table 1). The completion rate for questionnaires was 99.4% with only 39 persons not providing any further information.

**Table 1:** HIV testing and diagnosis rates (per 100,000 population) for persons seeking testing April 2013– March 2014 in Kiev City, Ukraine.

Characteristics		Population [17]	Number requesting HIV test *	HIV testing rate	Number newly diagnosed with HIV	Rate of New diagnoses
**Total**		2,172,448	6370	293.2	467	21.5
**Age (years)**	**16–25**	484,839	1466	302.4	64	13.2
	**26–30**	212,340	1795	845.3	120	56.5
	**31–35**	177,968	1456	818.1	154	86.5
	**≥36**	1,297,301	1653	127.4	129	9.9
**Sex**	**Male**	990,412	3425	345.8	303	30.6
	**Female**	1,182,036	2945	249.1	164	13.9

* Excluding 32 persons: 7 records were missing year of birth, sex or identification number, and 25 persons were already known to be HIV positive.

Probable route of exposure to HIV was available for 5986 of the 6017 (99.5%) individuals with a test result, the majority (4803; 80.2%) of whom reported heterosexual contact, with 191 (3.2%) identifying as MSM. Of the 4803 reporting heterosexual contact, 519 (10.8%) reported contact with PWIDs, with a higher proportion for women (417/2587, 16.1%) compared to men (101/2216, 4.6%). A further 377 (7.8%) reported paying for sex or reported that they themselves were paid for sex.

The median age among those newly-diagnosed was higher than those testing (32 years; IQR: 28–36), and HIV prevalence among those tested was highest among persons aged 31–35 years, males, PWID and MSM (Table 2). The diagnosis rate for PWID was estimated at 760.7 per 100,000 PWID and, for MSM, ranged from 516.3 to 1548.3 per 100,000 MSM. The proportion of persons newly-diagnosed among those in the heterosexual contact exposure category was low and similar for men and women. For PWID, the proportion newly-diagnosed was somewhat higher for women compared with men (21.5% vs. 17.1% respectively).

**Table 2:** Distribution of all persons testing for HIV and persons newly diagnosed at any one of the four HIV testing sites between April 2013 –March 2014 in Kiev City, Ukraine.

	Number tested for HIV	Number where test result available	Newly Diagnosed	
	N = 6370^≠^	N = 6017	N = 467	%
**Age (years)**				
≤25	1466	1381	64	4.6
26–30	1795	1698	120	7.1
31–35	1456	1376	154	11.2
≥36	1653	1562	129	8.3
**Sex**				
Male	3425	3224	303	9.4
Female	2945	2793	164	5.9
**Residence**				
Kiev (city)	5771	5457	391	7.2
Kiev area (small town)	412	384	53	13.8
Kiev area (village)	111	108	10	9.3
Another area of Ukraine	46	43	7	16.3
Not Reported	30	25	6	
**Probable route of exposure** *				
Persons who inject drugs: Male	835	801	137	17.1
Persons who inject drugs: Female	198	191	41	21.5
Men who have sex with men	201	191	46	24.1
Heterosexual contact: Male	2370	2216	114	5.1
Heterosexual contact: Female	2728	2587	122	4.7
Not reported	38	31	7	
**Testing history**				
Repeat tester	1413	1365	84	6.2
First-time tester	4787	4499	349	7.8
Not Reported	170	153	34	
**Reason for test** *				
Clinical Indicators	3166	2336	257	11.0
High Risk Behaviour	2427	2977	163	5.5
General Public Screening	735	668	40	6.0
Not Reported	42	36	7	

* Persons with more than one reported risk factor were classified by the following hierarchical order: PWID, MSM, then heterosexual contact. Persons with more than one reported reason for test were classified by the following hierarchical order: general Screening, clinical indication, then high risk population.

^**≠**^ Excluding 32 persons: 7 records were missing year of birth, sex or identification number, and 25 persons were already known to be HIV positive.

For 6200 individuals, information on testing history was available, of whom 1413 (23%) were identified as repeat testers. In univariate logistic regression models, repeat testers were more likely to be older, MSM or PWID, and where reason for test was high risk behaviour, compared with first time testers (Table 3). As the interaction between sex and probable route of exposure was found to be significant (p = 0.004), we re-categorised these two variables to create a new variable (MSM, female heterosexual contact, male heterosexual contact, female PWID, and male PWID).

**Table 3:** Factors associated with repeat testing among persons attending for an HIV test April 2013– March 2014 in Kiev City, Ukraine.

	Univariate		Multivariate*	
	OR (95% CI)	P value	OR (95% CI)	P value
**Age (per 10 year increase)**	1.21 (1.12–1.30)	<0.001	1.28 (1.19–1.39)	<0.001
**Area of residence**				
Kiev City	1		1	
Outside Kiev City	0.67 (0.54–0.85)	<0.001	0.66 (0.52–0.84)	<0.001
**Probable route of exposure**				
Heterosexual contact: Female	1		1	
Heterosexual contact: Male	0.81 (0.70–0.93)		0.78 (0.68–0.91)	
MSM	2.16 (1.59–2.93)	<0.001	1.87 (1.37–2.55)	<0.001
PWID: Female	1.52 (1.09–2.12)		1.40 (1.00–1.96)	
PWID: Male	2.10 (1.77–2.50)		1.93 (1.62–2.30)	
**Reason for test**				
Clinical Indicators	1		1	
General Pubic Screening	0.25 (0.19–0.34)	<0.001	0.28 (0.20–0.38)	<0.001
High Risk Behaviour	1.52 (1.34–1.72)		1.55 (1.37–1.76)	

* Adjusted for all other factors in table.

After adjusting for all other variables, probable route of exposure/sex remained an independent predictor for repeat testing. Compared to heterosexual women, MSM [Odds Ratio (OR): 1.87; 95% Confidence Interval (CI):1.37–2.55] and PWIDs (males and females) [OR = 1.93; 95% CI:1.62–2.30 and OR = 1.40; 95% CI:1.00–1.96, respectively] were more likely to repeat test, and heterosexual men were less likely to repeat test [OR = 0.78; 95% CI:0.68–0.91].

Furthermore, repeat testing was significantly more likely with increasing age [OR = 1.28 per 10 year increase, 95% CI:1.19–1.39] and among those engaging in high risk behaviour [OR = 1.55; 95% CI:1.37–1.76] compared with those testing due to clinical indicators. Repeat testing was less likely among those testing as part of screening [OR = 0.28; 0.20–0.38] and those resident outside the City [OR = 0.66; 95% CI:0.52–0.84] (Table 3).

Heterosexual women reporting high risk behaviour (high risk partner or sex work) had a higher proportion of repeat testers than heterosexual men overall (25%; 135/539 vs. 19%; 72/374), predominately led by women with partners who inject drugs (28%; 119/420).

## Discussion

We estimate an HIV prevalence of 7.8% for persons presenting for an HIV test in Kiev City, equivalent to 21.5 diagnoses per 100,000 population, and an overall testing rate of 293.2 per 100,000 population. For the first time detailed information on HIV risk behaviour was available, indicating that MSM and PWIDs were disproportionately affected by HIV. Furthermore, given the proportions among newly-diagnosed female heterosexuals reporting sexual contact with PWID there is further evidence of bridging in risk between PWID and their sexual partners.

Although, the diagnosis rate of 21.5 per 100,000 population in Kiev City is lower than that for Ukraine as a whole (37.1 per 100,000), it is however, substantially higher than rates for Western and Central Europe (6.6/100,000 and 2.1/100,000)[1]. Using MSM population estimates [13, 18], the diagnosis rate for MSM ranged from 490.6/100,000 to 1548.3/100,000. This is substantially higher than the diagnosis rates of 76.6 to 241.7/100,000 suggested by the 152 MSM diagnosed in Ukraine overall [1], though the prevalence of MSM may differ between Kiev and the rest of Ukraine.

Interestingly, there was little difference in the diagnosis rates between men and women reporting heterosexual contact. Given the high rates of stigma and discrimination within Ukraine we had anticipated non-disclosure of homosexuality to be high, with men more likely to report sex with women, leading to high HIV diagnosis rate in heterosexual men compared to heterosexual women. However, this was not reflected in the data, suggesting that any non-disclosed homosexuality among men is unlikely to be substantial. However, in agreement with previous findings, our data suggest a bridging between high risk individuals and the heterosexual population with an estimated 1 in 6 heterosexual women reporting contact with a PWID [8].

Furthermore, our data indicate an increasing number of women being diagnosed with HIV, particularly women who inject drugs who are at a higher risk as they are more likely to be second on the needle, exchange sex for drugs, subject to gender-based violence and stigma, and have unsafe sex with an injecting partner [19–21].

Testing patterns among persons presenting for an HIV test in Kiev City are consistent with the previous literature [22–27], where those at high risk of HIV were more likely to frequently test, both probable route of exposure (MSM and PWID) and reason for test (high risk behaviour) were independently associated with repeat testing. Age was also associated with repeat testing, which may, in part, be due to younger persons having had less time to test than those who are older. Around one in three MSM and PWID reported a previous test compared to one in five among heterosexuals, indicating that persons at risk have some awareness about testing; this was also evident among heterosexual women with partners who inject drugs.

It is important to bear in mind that data from Kiev City may well not be representative for the whole of Ukraine, particularly for regions with a lower HIV prevalence such as Chernivtsi, Ternopilm and Transcarpathian, and our findings may not be generalizable to the rest of the country. For a better understanding of the epidemic in other regions, we believe it would be beneficial to consider implementation of our methods across Ukraine. Acceptance rate was high (>99%) among attenders and information on HIV risk behaviour was also available for >99% of those completing the questionnaire. Population data for MSM was estimated using the published literature, based on work by the AIDS Alliance [14] or through the use of the European MSM internet survey (EMIS) [13]. There are definite uncertainties within these estimates, indicated by the range, and with both dependent on self-reported information, and further work is needed to establish the true numbers at risk.

This study provides more detailed information on persons testing and those newly diagnosed with HIV in Kiev City than had previously been available. It highlights the impact key populations have on the epidemic, feeding into current work on HIV prevention and response. We have shown that persons were comfortable with disclosing their risk, in particular men identifying as MSM. Diagnosis rates among MSM for Kiev City suggest that there may be a significant level of undisclosed homosexuality overall in Ukraine [9], further supporting work to minimise stigma and discrimination.

## Appendix

CASCADE Steering Committee: Julia Del Amo (Chair), Laurence Meyer (Vice Chair), Heiner C. Bucher, Geneviève Chêne, Osamah Hamouda, Deenan Pillay, Maria Prins, Magda Rosinska, Caroline Sabin, Giota Touloumi.

CASCADE Co-ordinating Centre: Kholoud Porter (Project Leader), Ashley Olson, Andrea Cartier, Lorraine Fradette, Sarah Walker, Abdel Babiker.

CASCADE Clinical Advisory Board: Heiner C. Bucher, Andrea De Luca, Martin Fisher, Roberto Muga

CASCADE Collaborators: Australia PHAEDRA cohort (Tony Kelleher, David Cooper, Robert Finlayson, Mark Bloch) Sydney AIDS Prospective Study and Sydney Primary HIV Infection cohort (Tony Kelleher, Tim Ramacciotti, Linda Gelgor, David Cooper, Don Smith); Austria Austrian HIV Cohort Study (Robert Zangerle); Canada South Alberta clinic (John Gill); Estonia Tartu Ülikool (Irja Lutsar); France ANRS CO3 Aquitaine cohort (Linda Wittkop, Francois Dabis, Rodolphe Thiebaut), ANRS CO4 French Hospital Database (Dominique Costagliola, Marguerite Guiguet), Lyon Primary Infection cohort (Philippe Vanhems), French ANRS CO6 PRIMO cohort (Marie-Laure Chaix, Jade Ghosn), ANRS CO2 SEROCO cohort (Laurence Meyer, Faroudy Boufassa); Germany German HIV-1 seroconverter cohort (Osamah Hamouda, Claudia Kücherer, Barbara Bartmeyer); Greece AMACS (Helen Sambatakou, Nikolaos V Sipsas, Charalambos A Gogos); Greek Haemophilia cohort (Giota Touloumi, Nikos Pantazis, Olga Katsarou); Italy Italian Seroconversion Study (Giovanni Rezza, Maria Dorrucci), ICONA cohort (Antonella d’Arminio Monforte, Andrea De Luca.) Netherlands Amsterdam Cohort Studies among homosexual men and drug users (Maria Prins, Ronald Geskus, Jannie van der Helm, Hanneke Schuitemaker); Norway Oslo and Ulleval Hospital cohorts (Mette Sannes, Oddbjorn Brubakk, Anne-Marte Bakken Kran, Anne Margarita Dyrhol Riise); Poland National Institute of Hygiene (Magdalena Rosinska); Spain Badalona IDU hospital cohort (Roberto Muga, Jordi Tor), Barcelona IDU Cohort (Patricia Garcia de Olalla, Joan Cayla), CoRIS-scv (Julia del Amo, Santiago Moreno, Susana Monge); Madrid cohort (Julia Del Amo, Jorge del Romero), Valencia IDU cohort (Santiago Pérez-Hoyos); Sweden Swedish InfCare HIV Cohort, Sweden (Anders Sönnerborg); Switzerland Swiss HIV Cohort Study (Heiner C. Bucher, Huldrych Günthard, Martin Rickenbach); Ukraine Perinatal Prevention of AIDS Initiative (Ruslan Malyuta); United Kingdom Public Health England (Gary Murphy), UK Register of HIV Seroconverters (Kholoud Porter, Anne Johnson, Andrew Phillips, Abdel Babiker), University College London (Deenan Pillay); African cohorts: Genital Shedding Study (US: Charles Morrison; Family Health International, Robert Salata, Case Western Reserve University, Uganda: Roy Mugerwa, Makerere University, Zimbabwe: Tsungai Chipato, University of Zimbabwe); International AIDS Vaccine Initiative (IAVI) Early Infections Cohort (Kenya, Rwanda, South Africa, Uganda, Zambia: Pauli N. Amornkul, IAVI, USA; Jill Gilmour, IAVI, UK; Anatoli Kamali, Uganda Virus Research Institute/Medical Research Council Uganda; Etienne Karita, Projet San Francisco, Rwanda).

EuroCoord Executive Board: Fiona Burns, University College London, UK; Geneviève Chêne (Chair), University of Bordeaux, France; Dominique Costagliola (Scientific Coordinator), Institut National de la Santé et de la Recherche Médicale, France; Carlo Giaquinto, Fondazione PENTA, Italy; Jesper Grarup, Region Hovedstaden, Denmark; Ole Kirk, Region Hovedstaden, Denmark; Laurence Meyer, Institut National de la Santé et de la Recherche Médicale, France; Heather Bailey, University College London, UK; Alain Volny Anne, European AIDS Treatment Group, France; Alex Panteleev, St. Petersburg City AIDS Centre, Russian Federation; Andrew Phillips, University College London, UK, Kholoud Porter, University College London, UK; Claire Thorne, University College London, UK.

EuroCoord Council of Partners: Jean-Pierre Aboulker, Institut National de la Santé et de la Recherche Médicale, France; Jan Albert, Karolinska Institute, Sweden; Silvia Asandi, Romanian Angel Appeal Foundation, Romania; Geneviève Chêne, University of Bordeaux, France; Dominique Costagliola, INSERM, France; Antonella d’Arminio Monforte, ICoNA Foundation, Italy; Stéphane De Wit, St. Pierre University Hospital, Belgium; Peter Reiss, Stichting HIV Monitoring, Netherlands; Julia Del Amo, Instituto de Salud Carlos III, Spain; José Gatell, Fundació Privada Clínic per a la Recerca Bíomèdica, Spain; Carlo Giaquinto, Fondazione PENTA, Italy; Osamah Hamouda, Robert Koch Institut, Germany; Igor Karpov, University of Minsk, Belarus; Bruno Ledergerber, University of Zurich, Switzerland; Jens Lundgren, Region Hovedstaden, Denmark; Ruslan Malyuta (Chair), Perinatal Prevention of AIDS Initiative, Ukraine; Claus Møller, Cadpeople A/S, Denmark; Kholoud Porter, University College London, United Kingdom; Maria Prins, Academic Medical Centre, Netherlands; Aza Rakhmanova, St. Petersburg City AIDS Centre, Russian Federation; Jürgen Rockstroh, University of Bonn, Germany; Magda Rosinska, National Institute of Public Health, National Institute of Hygiene, Poland; Manjinder Sandhu, Genome Research Limited; Claire Thorne, University College London, UK; Giota Touloumi, National and Kapodistrian University of Athens, Greece; Alain Volny Anne, European AIDS Treatment Group, France.

EuroCoord External Advisory Board: David Cooper, University of New South Wales, Australia; Nikos Dedes, Positive Voice, Greece; Kevin Fenton, Public Health England, USA; David Pizzuti, Gilead Sciences, USA; Marco Vitoria, World Health Organisation, Switzerland.

EuroCoord Secretariat: Silvia Faggion, Fondazione PENTA, Italy; Lorraine Fradette, University College London, UK; Richard Frost, University College London, UK; Andrea Cartier, University College London, UK; Dorthe Raben, Region Hovedstaden, Denmark; Christine Schwimmer, University of Bordeaux, France; Martin Scott, UCL European Research & Innovation Office, UK.
